# Supplementary material for: Deficiency of AP1 Complex Ap1g1 in Zebrafish Model Led to Perturbation of Neurodevelopment, Female and Male Fertility; New Insight to Understand Adaptinopathies
Source: Int J Mol Sci. 2023 Apr 12;24(8):7108. doi: 10.3390/ijms24087108 (PMC10138411; doi:10.3390/ijms24087108)
Supplement: Supplementary file 1 [file ijms-24-07108-s001.zip › Supplementary Figures rev 060423.pdf]

## Figure S1

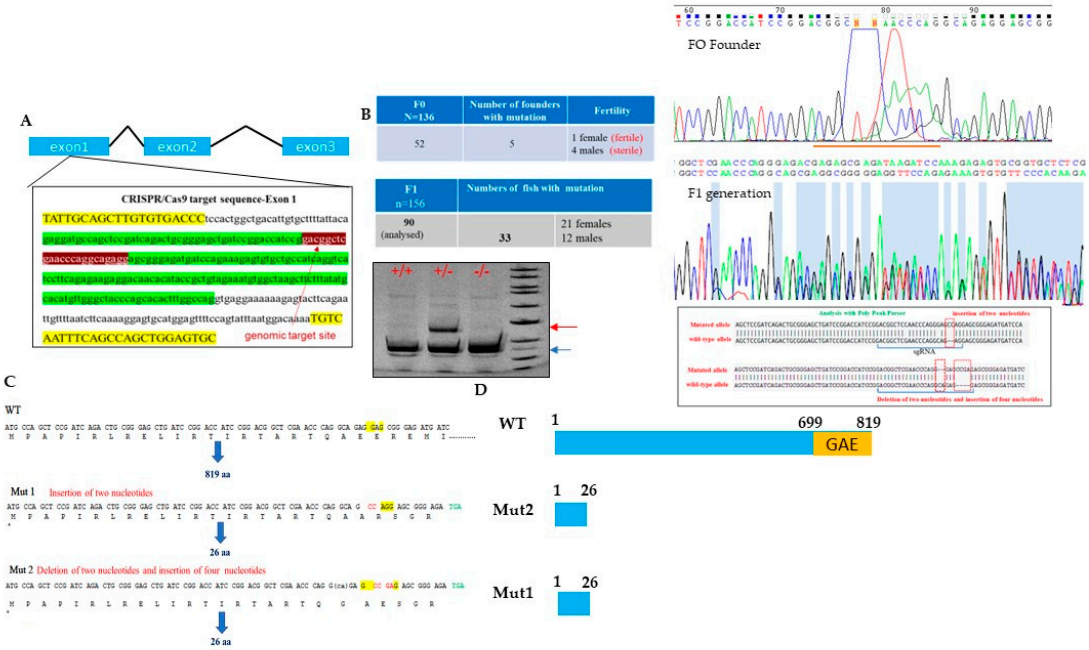


**Figure S1 Generation of γ1-adaptin knockout zebrafish using CRISPR/Cas9 targeting exon 1**

*(A) Selection of oligonucleotides to create ap1g1 sgRNA following the protocol of Gagnon et al. 2014. and using the CHOPCHOP algorithm. Exon 1 sequence is boxed in green, the sgRNA target sequence is boxed in brown and the sequences of forward and reverse primers used for screening are boxed in yellow. (B) Schematic representation of adult animals obtained as founders (F0) and F1 generation; chromatograms (Sanger Sequencing) of wild-type and heterozygous fish at 3 months post fertilization. The sgRNA target sequence is underlined in red F0 and F1 Chromatogram. In the boxed the gamma$^{+/+}$ (up) vs. gamma $^{-/-}$ (down) sequences, the CRISPR/Cas9-targeted site is in italic and the protospacer adjacent motif (PAM) is underlined. Schematic overview of PAGE-based (15%) genotyping for identification DNA fragments. Homoduplex and heteroduplex are easily identified based on their mobility rate and expected band of 360 bp is present in +/+, +/- and -/- embryos; the expected homoduplexes are indicated by blue arrow and heteroduplex are indicated by red arrow. Nucleotide and amino acid sequence showing the wild type and mutant lines; mutant 1: the insertion of two nucleotides (CC) and mutant 2: the insertion of four nucleotides is shown in red (CCAG). C) Ammino acid sequence of wild type and predicted mutated protein generated by CRISPR/Cas9. In both mutants, the generated stop codon is shown in green. Both mutations generated a frame shift and a truncated protein of 26 amino acids. D) Schematic representation of wild type and mutated proteins.*

## Figure S2

A) B)

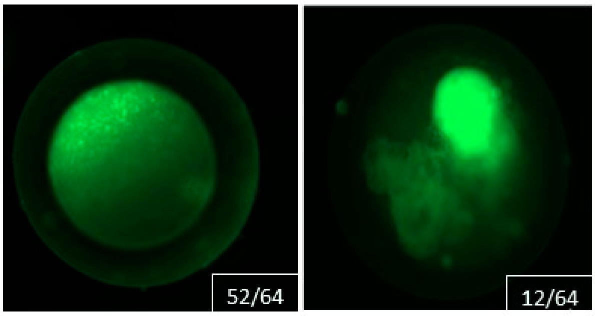

C)

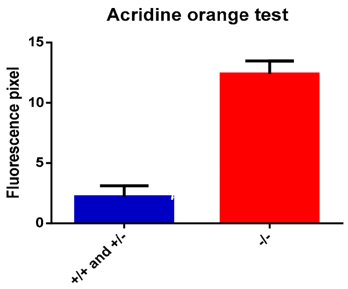


**Figure S2 Acridine orange staining for cells death**

*Dorsal view of embryos at 3.5 hpf stained with Acridine Orange (AO) flourescent dye to stain the incidence of apoptotic cell death in the +/+ and +/- embryos compare to -/- embryos. In A and B the numbers at the bottom of the images indicate the ratio between embryos with observed phenotype and the total number of stained embryos. (C) The graphs shows the quantification of AO fluorescence intensity expressed in pixels and obtained using ImageJ Software. Four experimental replicates represent the mean fluorescence intensity from each group sessions including at least 10 embryos*

## Supplementary Table S1

**Sequences of primers**

| Oligo name | SEQUENCE | |
|---|---|---|
| **Constant oligo** | AAAAGCACCGACTCGGTGCCACTTTTTCAAGTTGATAACGGACTAGCCTTATTT TAACTTGCTATTTCTAGCTCTAAAAC | |
| ***ap1g1* sg RNA** **site-specific oligo** | ATTTAGGTGACACTATA**GACGGCTCGAACCCAGGCAG**GTTTTAGAGCTAGAA ATAGCAAG | |
| *ap1g1For* *ap1g1Rev* | screening and sequencing | TATTGCAGCTTGTGTGACCC TGTCAATTTCAGCCAGCTGGAGTGC |
| *Dre rpl13aFor* *Dre rpl13aRev* | Real time PCR | TCTGGAGGACTGTAAGAGGTATGC AGACGCACAATCTTGAGAGCAG |
| *Dre BMP2b-Forw* *Dre BMP2b Rev* | Real time PCR | TCCATCACGAAGAAGCC ACTGACTTGTGTTCTGAG |
| *Dre BMP4-Forw* *Dre BMP4 Rev* | Real time PCR | AGCCAACACTGTGAGGAG TGTCTGGTGGAGGTGAGT |
| *Dre BMP7-Forw* *Dre BMP7 Rev* | Real time PCR | ACCTTCCATGTCAGCGTGTTC TGACCTTTCTGTTTGCCTCCTG |
| *Dre E-cadh-Forw* *Dre E cadh Rev* | Real time PCR | TCAGTACAGACCTCGACCGGCCAA AAACACCAGCAGAGAGTCGTAGG |
